# Lichen Planopilaris: Histopathological Study of Vertical Sections of Scalp Biopsies in 44 Patients

**Published:** 2012-08-30

**Authors:** Naser Tayyebi Meibodi, Fatemeh Asadi Kani, Yalda Nahidi, Jafar Bordbar Azari, Hamed Sadeghian

**Affiliations:** 1 Department of Pathology, Research Center for Skin Diseases and Cutaneous Leishmanaisis, Emam Reza Hospital, Faculty of Medicine, Mashhad University of Medical Sciences, Mashhad, IR Iran; 2 Department of Pathology, Shahid Beheshti University of Medical Sciences, Tehran, IR Iran

**Keywords:** Abdomen, Tuberculosis, Laparotomy

Dear Editor,

LPP is one of the main causes of primary cicatricial alopecias. This study was performed for review of histopathological characteristics of LPP, and for the first time the density of hair follicles in vertical scalp biopsies was compared with healthy scalp biopsies. Vertically sectioned scalp 5mm punch biopsies of 44 cases of LPP were examined(H & E and Alcian blue) according to NAHRS criteria [1]. Also we reviewed 22 age and sex matched scalp biopsies of autopsies for obtaining criteria for normal follicle number. We found normal values of hair follicles (15.24 ± 3.06), sebaceous glands (9.62 ± 2.29) and arrector pili muscles number (9.05 ± 2.55) in a 5 mm punch biopsy. Based on normal ranges, intensity of reduction in terminal hair was as follows: mild(9,12) moderate (5,8) and marked (1-4 follicles).

Characteristic findings of LPP were: markedly reduced hair density (63.6 %), absence of vellus hair (59.1 %), and follicular lichenoid changes (61.40 %). We found mucinous fibroplasias (50.0 %) and presence of interfollicular mucin (2.3 %). The only significant epidermal change was spongiosis (40.9 %). The most prominent pattern of follicular involvement was lichenoid (58.69%).Other changes included mild to moderate lymphocytic, primarily perifollicular (77.3 %) and perivascular (97.7 %) inflammation, Periinfundibular hypergranolosis (77.3 %), foreign body granuloma (13.6 %),demodex(25.0 %) , max-Josef cleft(38.6 %),epidermal(65.90 %) and follicular civatte bodies(45.45%). Vertical sections are useful in LPP in which the findings are focally confined to dermo-epidermal junction (DEJ) and superficial dermis [2].

Common findings in LPP are as follows: lichenoid lymphocyte infiltration in follicular DEJ [3][4][5], wedge shaped hypergranolosis [3][5], Colloid bodies [5], loss of sebaceous glands and destruction of hair follicle root sheaths [3][6][7] and follicular plugging [5]. In late lesions, lamellar perifollicular fibrosis is seen around isthmus, and finally the follicles are completely substituted with fibrous tracts [3][5]. Lichenoid infiltrate disappears [5]. Clefts may be seen between follicular epithelium and the dermis around it [5]. In our study, decrease or lack of terminal hair was seen in 93.1% and vellus hair in 59.1%, arrector pili decrease in 36%, its lack in 9.1%, reduction in sebaceous glands in 36% and its lack in 52%. In Tandon study, these findings were in 100%, 96%, 59%, 19%, 30% and 70% respectively [4].

In LPP, lichenoid changes are seen more than vacuolar degeneration [8]. In our study, follicular epithelium changes were lichenoid in 59.13%, spongiotic in 18.18% and vacuolar in 2.27%. In Tandon study, the most common follicular involvement was lichenoid (22%) and spongiotic (15%) [4]. On the contrary, in interfollicular epidermis in our samples, vacuolar changes (31.81%) were higher than lichenoid(18.81%) while Tandon has found epidermal involvement to be lichenoid in 7% and vacuolar in 4% [4]. Parakeratosis was seen in 13.6%, hyperkeratosis in 68.18% and follicular plugging in 72.7%. In Mehrgan’s study follicular plugging has been mentioned as an auxiliary finding in LPP (in 59%) and parakeratosis was not common.9 In Tendon study, prevalence of parakeratosis, hyperkeratosis and follicular plugging was 15%, 4% and 11%. It may be due to more advanced disease in his study [4].

Epidermal and follicular cytoid bodies were seen in 66% and 45% of our patients. Mehrgan has reported the cytoid body prevalence to be 53% [9]. In LPP, inflammation is mainly lymphocytic, and in early stages, infindibulum and isthmus of hair are afflicted [10]. Inflammation intensity in our patients was mild in 45.5 %, moderate in 38.4 % and severe in 15.9 % and was perifollicular in 77.3 % and perivascular in 97.7 % of cases. In Tandon study, inflammation intensity and location was almost similar to ours.[4] Unlike DLE, mucin is not seen in interfollicular dermis in LPP [3][10], but there was interfollicular mucin in one of our patients .There may be interfollicular mucin in LPP especially in case of overlap with DLE. Mucinous fibroplasias and perifollicular lamellar fibrosis was seen in 50% and 15.9% of our patients. These changes were seen in 37% and 11% of cases in Tandon study [4]. In this study, for the first time a criterion was presented for intensity of alopecia in vertical sections. We recommend a prospective study on cases of LPP with DIF microscopy and mucin stainig.
